# *N*-Methylolchloracetamid: MAK-Begründung, Nachtrag

**DOI:** 10.34865/mb283219kskd10_2ad

**Published:** 2025-06-30

**Authors:** Andrea Hartwig, MAK Commission

**Affiliations:** 1 Institut für Angewandte Biowissenschaften. Abteilung Lebensmittelchemie und Toxikologie. Karlsruher Institut für Technologie (KIT) Adenauerring 20a, Geb. 50.41 76131 Karlsruhe Deutschland; 2 Ständige Senatskommission zur Prüfung gesundheitsschädlicher Arbeitsstoffe. Deutsche Forschungsgemeinschaft, Kennedyallee 40, 53175 Bonn, Deutschland. Weitere Informationen: Ständige Senatskommission zur Prüfung gesundheitsschädlicher Arbeitsstoffe | DFG

**Keywords:** N-Methylolchloracetamid, Nase, Reizwirkung, Kanzerogenität, Formaldehydabspalter, Keimzellmutagenität, Sensibilisierung, N-methylolchloroacetamide, nose, irritation, carcinogenicity, formaldehyde releaser, germ cell mutagenicity, sensitization

## Abstract

The German Senate Commission for the Investigation of Health Hazards of Che﻿mi﻿cal Compounds in the Work Area (MAK Commission) has re-evaluated *N*-﻿methy﻿lolchloroacetamide [2832-19-1] with regard to its carcinogenicity and germ cell mutagenicity classification, its ability to be absorbed through the skin, its sensitization potential and whether an occupational exposure limit value (maximum concentration at the workplace, MAK value) can be derived. Relevant studies were identified from a literature search. The substance is a formaldehyde releaser and is expected to rapidly decompose in aqueous solution. For this reason, local irritation is expected and attrib﻿uted to the products formaldehyde and chloroacetamide. Carcinogenicity, toxicity and genotoxicity of *N*-methylolchloroacetamide in the upper respiratory tract or nose, the likely target organs, have not been investigated. The substance has mutagenic and clastogenic potential in vitro, presumably due to the release of formaldehyde. Formaldehyde was classified in Carcinogen Category 4 because it induces tumours in nasal tissue at concentrations that exceed its detoxification capacity. As a formaldehyde releaser, *N*-﻿methy﻿lolchloroac﻿etamide could likewise be classified in Carcinogen Category 4. However, because it is not possible to derive a MAK value for *N*-methylolchloroacetamide, the substance has been assigned to Carcinogen Category 2 and given the footnote “Prerequisite for Cat﻿egory 4 in principle fulfilled, but insufficient data available for the establishment of a MAK or BAT value”. As there are no data on the systemic bioavailability of *N*-methy﻿lolchloroacetamide and the formaldehyde that is released in tissues, there is no experimental evidence that the formaldehyde reaches the germ cells. Therefore, *N*-﻿methy﻿lolchloroacetamide has been classified in Category 3 B for germ cell mutagens. *N*-﻿Methy﻿lolchloroacetamide has a skin sensitizing potential. Skin contact is not expected to contribute significantly to systemic toxicity.

**Table:** 

**MAK-Wert**	**–**
**Spitzenbegrenzung**	**–**
	
**Hautresorption**	**–**
**Sensibilisierende Wirkung (1992)**	**Sh**
**Krebserzeugende Wirkung (2024)**	**Kategorie 2^a)^**
**Fruchtschädigende Wirkung**	**–**
**Keimzellmutagene Wirkung (2024)**	**Kategorie 3 B**
	
**EKA**	**–**
	
Synonyma	Chloracetamid-*N*-methylol 2-Chlor-*N*-hydroxymethylacetamid *N*-Hydroxymethylchloracetamid
Chemische Bezeichnung (IUPAC-Name)	2-Chlor-*N*-(hydroxymethyl)acetamid
CAS-Nr.	2832-19-1
Formel	H_2_CCl–CO–NH–CH_2_OH
	C_3_H_6_ClNO_2_
Molmasse	123,54 g/mol
Dampfdruck bei 25 °C	5,36 × 10^–7^ – 0,002 hPa (ber.; US EPA 2023)
log K_OW_	–1,58 bis –0,985 (ber.; US EPA 2023)
	
Hydrolysestabilität	langsamer Zerfall zu Formaldehyd und 2-Chloracetami﻿d in wässriger Lösung (k. w. A.; Henschler 1992)
Einsatzverbote	Der Stoff ist unter REACH lediglich vorregistriert (ECHA 2023) und wird nach Angabe von Biozidherstellern in Deutschland nicht mehr in Kühlschmierstoffen eingesetzt (Hartwig und MAK Commission 2023 a).

a) Voraussetzung für Kategorie 4 prinzipiell erfüllt, aber Daten für MAK- oder BAT-Wert-Ableitung nicht ausreichend

Hinweis: Formaldehydabspalter

Es liegt eine Begründung aus dem Jahr 1992 vor (Henschler 1992). Es erfolgt eine Neubewertung der Daten zur Formaldehydabspaltung nach aktuellem Vorgehen der Kommission (DFG 2024). Unter diesem Aspekt werden in diesem Nachtrag nur die bewertungsrelevanten Endpunkte reevaluiert.

Über die Stabilität und Zerfallskinetik liegen keine verlässlichen Angaben vor, außer dass der Stoff langsam zerfällt, wobei aus einem Molekül *N*-Methylolchloracetamid jeweils ein Molekül Formaldehyd und 2-Chloracetamid entstehen.

## Allgemeiner Wirkungscharakter

Der vermutlich kritische Effekt von *N*-Methylolchloracetamid ist die kanzerogene und reizende Wirkung von freigesetztem Formaldehyd am Atemtrakt. Im Tierversuch weist die Substanz eine geringe akute Toxizität auf. Über Reizeffekte an Haut und Augen ist nichts bekannt. *N*-Methylolchloracetamid kann sensibilisierend wirken. Während der Stoff an Salmonella typhimurium mit und ohne metabolische Aktivierung nicht mutagen ist, ergibt die Untersuchung an E. coli einen positiven Befund. An menschlichen Lymphozyten in vitro ist im Chromosomenaberrationstest eine schwache klastogene Wirkung festzustellen. Im Mikronukleustest an der Maus ist *N*-Methylolchloracetamid bei intraperitonealer Applikation klastogen, jedoch nicht nach oraler Gabe. Es liegen keine Untersuchungen zur kanzerogenen Wirkung vor.

## Wirkungsmechanismus

*N*-Methylolchloracetamid ist ein Formaldehydabspalter.

Formaldehyd zeigt deutliche Reizwirkungen in der Nase und kann bei chronischer Exposition Tumoren im Epithel der Nasenschleimhaut erzeugen. Die Entgiftungskapazitäten des Nasengewebes für Formaldehyd werden bei einem MAK-Wert von 0,3 ml/m^3^ (0,37 mg/m^3^) nicht überschritten (Greim 2000; Hartwig 2010).

Der berechnete Dampfdruck von *N*-Methylolchloracetamid (5,36 × 10^–7^ bis 0,002 hPa) zeigt, dass der Stoff bei Konzentrationen, bei denen Formaldehyd schon dampfförmig ist (Dampfdruck 5185 hPa; OECD 2002), noch als Aerosol vorliegt. Für Aerosole von Formaldehydabspaltern ist durch die Impaktierung im Atemtrakt mit einer stärkeren Wirkung als der durch dampfförmigen Formaldehyd verursachten Wirkung zu rechnen, wie mit einem anderen Formaldehydabspalter, zu dem eine Inhalationsstudie vorliegt, gezeigt wurde (siehe Begründung *N*,*N′*,*N′′*-Tris(β-hydroxyethyl)hexahydro-1,3,5-triazin; Hartwig und MAK Commission 2023 b).

## Toxikokinetik und Metabolismus

In Sensibilisierungstests wurde als Höchstkonzentration 1 % eingesetzt (Henschler 1992), es ist daher davon auszugehen, dass dies einer nicht mehr reizenden Lösung entspricht. Für eine 1%ige wässrige Lösung und einem log K_OW_ von –1,58 berechnen sich daraus mit dem Modell von Fiserova-Bergerova et al. (1990) und dem Algorithmus des IH SkinPerm-Modells (Tibaldi et al. 2014) Fluxe von 3,9 bzw. 0,869 µg/cm² und Stunde. Unter der Annahme einer einstündigen Exposition von 2000 cm² Hautoberfläche würde dies Aufnahmemengen von 7,8 bzw. 1,7 mg entsprechen.

## Kanzerogenität

Hierzu liegen keine Daten vor.

## Bewertung 

Kritische Effekte sind die kanzerogene und lokal reizende Wirkung des abgespaltenen Formaldehyds.

**Krebserzeugende Wirkung. **Es liegen keine Untersuchungen der krebserzeugenden Wirkung von *N*-Methylolchloracetamid vor. *N*-Methylolchloracetamid selbst weist eine schwache klastogene Potenz auf, eine mögliche genotoxische Wirkung am wahrscheinlichen Zielgewebe Nase (wie bei Formaldehyd) ist jedoch nicht untersucht.

2-Chloracetamid ist nicht genotoxisch (Hartwig 2012), eine kanzerogene Wirkung daher unwahrscheinlich.

Die lokale Kanzerogenität des Hydrolyseprodukts Formaldehyd hingegen ist ausführlich dokumentiert (Greim 2000; Hartwig 2010). Formaldehyd ruft Nasentumoren hervor, jedoch erst bei Konzentrationen, die die Entgiftungskapazitäten des Nasengewebes überschreiten. Daher ist Formaldehyd in Kanzerogenitäts-Kategorie 4 eingestuft.

Aufgrund der lokalen kanzerogenen Wirkung von Formaldehyd könnte *N*-Methylolchloracetamid in Analogie zu Formaldehyd in Kanzerogenitäts-Kategorie 4 eingestuft werden. Da jedoch kein MAK-Wert für *N*-Methylolchloracetamid abgeleitet werden kann, wird der Stoff der Kanzerogenitäts-Kategorie 2 zugeordnet und erhält die Fußnote „Voraussetzung für Kategorie 4 prinzipiell erfüllt, aber Daten für MAK- oder BAT-Wert-Ableitung nicht ausreichend“.

**MAK-Wert. **Es liegen keine Inhalationsstudien mit *N*-Methylolchloracetamid am Menschen oder am Tier vor, aus denen ein MAK-Wert abgeleitet werden kann.

Wegen des Fehlens genauer Daten wird als Worst-Case-Abschätzung davon ausgegangen, dass *N*-Methylolchloracetamid beim Auftreffen im Atemtrakt sofort vollständig zu Formaldehyd und 2-Chloracetamid zerfällt und der Abbau des aus *N*-Methylolchloracetamid entstehenden Formaldehyds langsamer erfolgt als dessen Bildung.

Beim Auftreffen im Atemtrakt ist mit einer inhalationstoxischen Wirkung durch Formaldehyd und 2-Chloracetamid zu rechnen.

Zu 2-Chloracetamid liegt eine MAK-Begründung vor. Aufgrund einer fehlenden Inhalationsstudie und da Reizwirkungen an Haut und Auge gezeigt wurden, konnte kein MAK-Wert abgeleitet werden (Hartwig 2012). Formaldehyd hat einen MAK-Wert von 0,3 ml/m^3^ (0,37 mg/m^3^), der vor der lokalen kanzerogenen Wirkung schützt und aus der augenreizenden Wirkung abgeleitet ist (Greim 2000; Hartwig 2010). Sein Dampfdruck bei 25 °C beträgt 5185 hPa (OECD 2002).

Der berechnete Dampfdruck von *N*-Methylolchloracetamid (5,36 × 10^–7^ bis 0,002 hPa) zeigt, dass der Stoff als Aerosol vorliegt. Für Aerosole von Formaldehydabspaltern ist durch die Impaktierung im Atemtrakt mit einer stärkeren Wirkung als die durch dampfförmiges Formaldehyd verursachte zu rechnen, wie mit einem anderen Formaldehydabspalter, zu dem eine Inhalationsstudie vorliegt, gezeigt wurde (siehe Begründung *N*,*N′*,*N′′*-Tris(β-hy﻿droxyethyl)hexahy﻿dro-1,3,5-triazin; Hartwig und MAK Commission 2023 b). Ein MAK-Wert in Analogie zu Formaldehyd kann daher nicht abgeleitet werden. Da auch keine NOAEC aus einer Inhalationsstudie vorliegt, kann für *N*-Methylolchloracetamid kein MAK-Wert festgesetzt werden. Eine Spitzenbegrenzung entfällt.

Bei Anwendung in verdünnten wässrigen Lösungen sollte mit einer Abspaltung von Formaldehyd gerechnet und daher der MAK-Wert für Formaldehyd (Greim 2000; Hartwig 2010) eingehalten werden.

**Keimzellmutagene Wirkung. ***N*-Methylolchloracetamid wirkt an Salmonella typhimurium mit und ohne metabolische Aktivierung nicht mutagen, jedoch an E. coli. An menschlichen Lymphozyten im Chromosomenaberrationstest in vitro ist der Stoff schwach klastogen. Im Mikronukleustest an der Maus ist *N*-Methylolchloracetamid bei intraperitonealer Applikation positiv, nach oraler Gabe nicht (Henschler 1992). Diese genotoxischen Effekte stimmen mit den für Formaldehyd beobachteten überein. Untersuchungen an Keimzellen liegen mit *N*-Methylolchloracetamid nicht vor.

Formaldehyd ist in Kategorie 5 für Keimzellmutagene eingestuft. Dies bedeutet, dass bei Einhaltung des MAK-Wertes von 0,3 ml/m^3^ nur ein sehr geringer Beitrag zum genetischen Risiko für den Menschen zu erwarten ist (Greim 2000).

Methylolchloracetamid könnte zwar in Analogie zu Formaldehyd in die Kategorie 5 für Keimzellmutagene eingestuft werden, jedoch kann für *N*-Methylolchloracetamid kein MAK-Wert aufgestellt werden.

Da Daten zur systemischen Bioverfügbarkeit des *N*-Methylolchloracetamids und dem freigesetzten Formaldehyd fehlen, liegt kein experimenteller Beleg vor, dass der freigesetzte Formaldehyd in aktiver Form die Keimzellen erreicht. Daher wird *N*-Methylolchloracetamid in Kategorie 3 B für Keimzellmutagene eingestuft.

**Fruchtschädigende Wirkung. **Es liegen keine Daten zur fruchtschädigenden Wirkung vor. Da kein MAK-Wert aufgestellt werden kann, entfällt die Zuordnung zu einer Schwangerschaftsgruppe.

**Hautresorption. **Zur dermalen Aufnahme von *N*-Methylolchloracetamid liegen keine experimentellen Daten vor. Abschätzungen auf der Basis von Modellrechnungen lassen unter Standardbedingungen (eine Stunde Exposition, 2000 cm^2^ eine Resorption von maximal 7,8 mg für eine nicht reizende 1%ige Lösung erwarten. Ein systemischer NOAEL für *N*-﻿Methylolchloracetamid zum Vergleich kann aus der vorhandenen Datenbasis nicht abgeleitet werden. Eine Markierung mit „H“ kann daher mit diesen Daten nicht erfolgen.

*N*-Methylolchloracetamid zerfällt jedoch zu je einem Mol Formaldehyd und 2-Chloracetamid. Unter der Worst-Case-Annahme eines vollständigen Zerfalls von *N*-Methylolchloracetamid mit Bildung von Formaldehyd lässt sich die Zunah﻿me des Formaldehydspiegels im Blut nach dermaler Applikation einer 1%igen wässrigen Lösung von *N*-﻿Methy﻿lolchloracetamid unter Standardbedingungen abschätzen: aus den oben vorhergesagten maximal dermal aufgenommenen 7,8 mg Methylolchloracetamid (0,0628 mmol) werden 1,89 mg Formaldehyd in einer Stunde freigesetzt, pro Minute demnach etwa 31,4 µg bzw. 39,3 µg/1,25 Minuten (mittlere Halbwertszeit des Formaldehyds im Blut; Kaden et al. 2010). Die transdermale Aufnahme von Methylolchloracetamid in den letzten sechs Halbwertszeit-Intervallen führt demnach zu einer im Blut zirkulierenden Formaldehydmenge von (39,3 + 19,7 + 9,8 + 4,9 + 2,5 + 1,2) µg = 77,4 µg. Der physiologisch bedingte Formaldehydspiegel im Blut des Menschen beträgt etwa 2–3 mg/l (10–15 mg in 5 Liter Blut, frei und reversibel gebunden) (Heck et al. 1985). Der zusätzliche Eintrag durch transdermal aufgenommenes Methylolchloracetamid erhöht demnach die Gleichgewichtskonzentration von Formaldehyd im Blut unbedeutend, sodass der Stoff weiterhin nicht mit „H“ markiert wird.

In einem weiteren Ansatz kann die „H“-Markierung auch unter dem Blickpunkt des zweiten Produkts 2-Chloracetamid betrachtet werden, dieses ist mit „H“ markiert. Unter der bereits dargestellten Worst-Case-Annahme eines raschen und vollständigen Zerfalls von *N*-Methylolchloracetamid mit Bildung von 2-Chloracetamid lässt sich die Zunahme des Spiegels von 2-Chloracetamid im Blut nach dermaler Applikation einer 1%igen wässrigen Lösung von *N*-Methy﻿lolchloracetamid unter Standardbedingungen abschätzen: Aus den oben vorhergesagten maximal dermal aufgenommenen 7,8 mg *N*-Methylolchloracetamid (0,0628 mmol) werden 5,87 mg 2-Chloracetamid in einer Stunde freigesetzt. Dies wür﻿de 0,084 mg/kg KG (bei 70 kg Körpergewicht) entsprechen.

Für 2-Chloracetamid wird ein NOAEL der Ratte von 10 mg/kg KG und Tag für Hodentoxizität aus einer 90-Tage-Fütterungsstudie angenommen (vgl. Hartwig 2012), dies entspricht beim Menschen 1,75 mg/kg KG und Tag mit Berücksichtigung der Übertragung der Ergebnisse aus dem Tierversuch auf den Menschen (1:2). Wegen der Hodentoxizität ist bei einer 90-Tage-Studie keine Zeitextrapolation nötig. Die Aufnahme von 2-Chloracetamid aus dem Zerfall von dermal penetrierter 1%iger Lösung von *N*-Methylolchloracetamid liegt damit deutlich unter der systemisch tolerablen Menge, und der Stoff wird auch aufgrund dieser Abschätzung nicht mit „H“ markiert.

**Sensibilisierende Wirkung. **Es gibt anhand der aktuellen Datenlage keine Handhabe für eine Änderung der Markierung. Die Markierung mit „Sh“ wird beibehalten, es erfolgt weiterhin keine Markierung mit „Sa“.
